# Epithelial rotation is preceded by planar symmetry breaking of actomyosin and protects epithelial tissue from cell deformations

**DOI:** 10.1371/journal.pgen.1007107

**Published:** 2017-11-27

**Authors:** Ivana Viktorinová, Ian Henry, Pavel Tomancak

**Affiliations:** Max Planck Institute of Molecular Cell Biology and Genetics, Dresden, Germany; Fred Hutchinson Cancer Research Center, UNITED STATES

## Abstract

Symmetry breaking is involved in many developmental processes that form bodies and organs. One of them is the epithelial rotation of developing tubular and acinar organs. However, how epithelial cells move, how they break symmetry to define their common direction, and what function rotational epithelial motions have remains elusive. Here, we identify a dynamic actomyosin network that breaks symmetry at the basal surface of the *Drosophila* follicle epithelium of acinar-like primitive organs, called egg chambers, and may represent a candidate force-generation mechanism that underlies the unidirectional motion of this epithelial tissue. We provide evidence that the atypical cadherin Fat2, a key planar cell polarity regulator in *Drosophila* oogenesis, directs and orchestrates transmission of the intracellular actomyosin asymmetry cue onto a tissue plane in order to break planar actomyosin symmetry, facilitate epithelial rotation in the opposite direction, and direct the elongation of follicle cells. In contrast, loss of this rotational motion results in anisotropic non-muscle Myosin II pulses that are disorganized in plane and causes cell deformations in the epithelial tissue of *Drosophila* eggs. Our work demonstrates that atypical cadherins play an important role in the control of symmetry breaking of cellular mechanics in order to facilitate tissue motion and model epithelial tissue. We propose that their functions may be evolutionarily conserved in tubular/acinar vertebrate organs.

## Author summary

Movement of epithelial tissues is essential for organ and body formation as well as function. To facilitate epithelial movements, cells need an internal or external source of mechanical force and a collective decision in which direction to move. However, little is known about the underlying mechanism of collective cell movement in living and moving epithelial tissues. Using high-speed confocal imaging of rotating follicle epithelia in acinar-like *Drosophila* egg chambers, we find that individual cells polarize their actomyosin network, a potent force-generating source, at their basal surface. We show that the atypical cadherin Fat2, a key regulator of planar cell polarity in *Drosophila* oogenesis, unifies and amplifies the polarized non-muscle Myosin II of individual follicle cells to break the symmetry of actomyosin contractility at the epithelial level. We propose that this is essential to facilitate epithelial rotation, and thereby directed cell elongation, at the basal surface of follicle cells. In contrast, a lack of unidirectional actomyosin contractility results in disrupted non-muscle Myosin II polarity within follicle cells and causes asynchronous Myosin II pulses that deform follicle cells. This demonstrates the critical function of Fat2, in the planar symmetry breaking of actomyosin, in epithelial motility, and potentially in organ development.

## Introduction

**Fig 1:**
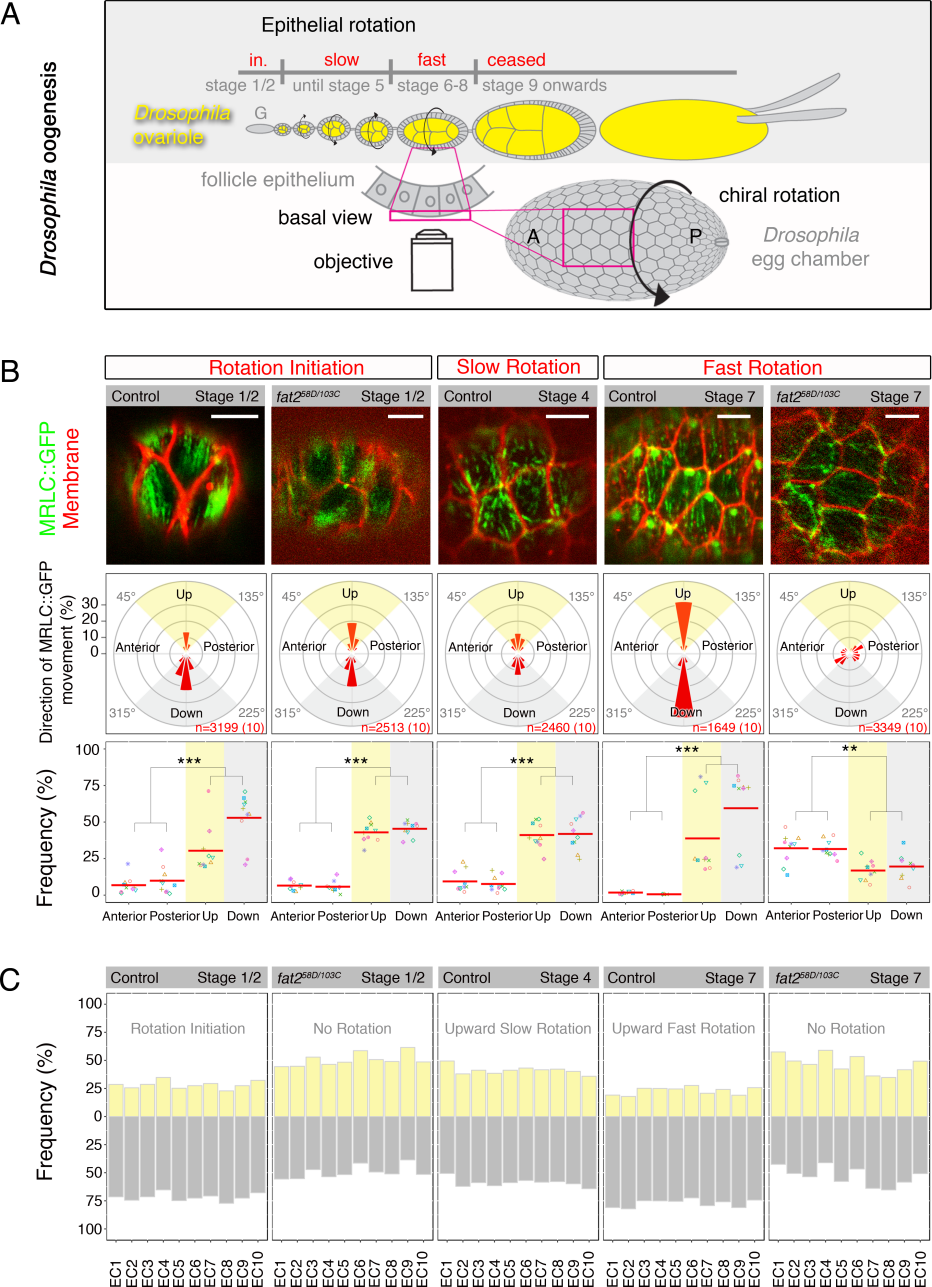
Planar symmetry breaking of Myo-II precedes epithelial rotation in Fat2-dependent manner. **(A)** *Drosophila* ovaries consist of ovarioles, which contain egg chambers of different stages. They bud from the germarium (G) and undergo a phase of rotation initiation (in.), followed by slow (clockwise or anti-clockwise, i.e. chiral) epithelial rotation (until stage 5), the speed of rotation accelerates during stages 6–8 and rotation ceases at stage 9. The confocal view of the basal surface of the follicle epithelium, covering nurse cells and the oocyte (yellow), that was analyzed is indicated. A = anterior, P = posterior. **(B)** First row: MRLC::GFP localization (green) at the basal surface of the follicle epithelium during rotation initiation (stage 1/2), slow (stage 4), fast (stage 7) and no epithelial rotation (*fat2* mutant stage 1/2 and 7) of *Drosophila* egg chambers. Scale bars = 5μm. Anterior is on the left. Middle row: Angular distribution of MRLC::GFP movement is shown in 20 degree-bin rose diagrams. The numbers of MRLC::GFP individual signals and egg chambers (in brackets) analyzed is at the lower right in and shown in red. 0° represents MRLC::GFP movements towards the anterior. The fraction of MRLC::GFP signal movements in a bin as a percentage of all MRLC::GFP movements analyzed is indicated. Anterior (315°≤45°), Posterior (135°≤225°), Up (45°≤135°, yellow) and Down (225°≤315°, grey) quadrants are shown. Last row: Fractions of MRLC::GFP movement within an image frame over time (Material and Methods) are shown for indicated quadrants and individual egg chambers (displayed by different colour and shape). Note that MRLC::GFP moves preferentially in Up and Down quadrants during rotation initiation along with slow and fast rotation controls, and this directionality is lost in *fat2* mutant egg chambers. Mean (red bar) and *P-*values < 0.001 (***) and <0.01 (**) are shown. **(C)** Frequencies of MRLC::GFP movement of Up (yellow) and Down (grey) quadrants for 10 individual egg chambers (EC) when the direction of the epithelial rotation is unified towards Up (Material and Methods).

Functional organ morphogenesis [1–3] has been linked to turns and rotations of epithelial sheets [4–9] relative to the organ or body anterior-posterior (AP) axis. The primary determinant of this chirality has been associated with the cytoskeleton in different species [10–14]. In rotating *Drosophila* organs such as the hindgut [9] and male genitalia [6], the consistent handedness of epithelial rotation depends on *myosinID (myoID)* and utilizes asymmetric cellular intercalations. An alternative form of rotational movement has been recently identified in the *Drosophila* ovary [8]. Here, organ-like structures, called egg chambers, display rotation of an edgeless monolayered follicle epithelium together with underlying germline cells (called nurse cells and the oocyte) that all rotate along the surrounding rigid extracellular matrix (ECM), called the basement membrane (BM) [8] (Fig 1A). In contrast to the *Drosophila* hindgut and male genitalia where cell membranes adopt a specific form of asymmetry called planar cell chirality (PCC), the follicle epithelium displays no apparent membrane PCC (S1A Fig, Material and Methods), and different egg chamber units in one animal can rotate clockwise or anti-clockwise performing more than three full rotations around their AP axis during early and mid-oogenesis [8, 15]. This suggests that an alternative, possibly *myoID*-independent, mechanism drives this collective cell behaviour. Interestingly, the basal surface of each follicle cell displays clear local chirality of actin-rich protrusions and chiral localization of several planar cell polarity (PCP) molecules that are genetically implicated in egg chamber rotation [16–23]. Epithelial rotation is initially slow during early oogenesis (stages 2/3-5: average speed ~ 0.2 μm/min) [24], accelerates in mid-oogenesis (stages 6–8: average speed ~ 0.5–0.6 μm/min) [8, 18, 20, 24] and stops at stage 9 [8]. It has been shown that microtubule (MT) growth predicts the direction of epithelial rotation in early and mid-oogenesis and that their planar symmetry breaking during rotation initiation (stage 1/2) is regulated by the atypical cadherin Fat2 [24, 25]. Fat2 is a key PCP regulator of the actin cytoskeleton [25] as well as BM components [16, 19, 26] and is required for the epithelial rotation and elongation of *Drosophila* egg chambers [20, 25]. In addition, the Fat2 planar polarized (zig-zag) pattern at the basal lagging membrane surface of follicle cells depends on MTs during fast epithelial rotation [20]. There is no evidence that MTs represent the active force-generating mechanism that drives epithelial rotation, which recently has been shown to involve actin-rich protrusions [18, 23]. However, non-muscle myosin II (Myo-II), which generally provides contractility and force generation to the actin cytoskeleton, is missing on actin-rich protrusions [18]. Therefore, motivated by the observation that pharmacological depletion of ROCK activity (Rho kinase inhibitor) leads to no epithelial rotation [20], we hypothesized that the basal actin filaments containing Myo-II are better candidates to fulfill this force generating function. To test this hypothesis we investigated the function of Myo-II, its connection to the PCP pathway in *Drosophila* epithelial rotation, and the role of their interplay in this epithelial tissue.

## Results

### Myo-II behaviour is highly dynamic at the basal surface of the follicle epithelium

In order to understand the function of Myo-II in epithelial rotation, we employed *ex vivo* high-speed confocal live imaging to observe the behaviour of Myo-II regulatory light chains (MRLC, called Spaghetti Squash, *sqh* in *Drosophila*) at the basal surface of the follicle epithelium. In order to image the MRLC, we used a MRLC fusion protein (MRLC::GFP) and imaged in a null *sqh*^*AX3*^ mutant [27] to avoid competition with endogenous Myo-II.

Using this method, we uncovered a very dynamic ‘dot-like’ pattern of Myo-II with an average size of 363 nm ± 0.05 nm (n = 136) in a thin layer (≤1000 nm) at the basal surface of the rotating follicle epithelium (control stage 1/2, control stage 4 and control stage 7) as well as in static *fat2*^*58D/103C*^ mutant egg chambers (stages 1/2 and 7), which have been previously shown to lack epithelial rotation [18, 20] (Fig 1B first row and S1–S5 Movies). Next, we calculated the average speed of MRLC::GFP movement in rotating egg chambers at the time of rotation initiation (2.1 μm/min ± 0.71 (n = 80) for control stage 1/2), slow (2.11 μm/min ± 0.79 (n = 101) for control stage 4) and fast (2.44 μm/min ± 0.96 (n = 105) for control stage 7) epithelial rotation. We found that these average speeds of MRLC::GFP movement did not significantly differ (*P* = 0.11) from the average speeds of MRLC::GFP movement in static *fat2* mutant egg chambers (2.23 μm/min ± 0.49 (n = 81) for stage 1/2 and 1.99 μm/min ± 0.62 (n = 100) for stage 7). In addition, we identified large intense MRLC::GFP dots (1.01um ± 0.14 um, n = 50), which were close to the lagging end of migrating follicle cells during fast epithelial rotation (Fig 1B first row and S3 Movie) and lost in the static *fat2* mutant follicle epithelium of corresponding stage (Fig 1B first row and S5 Movie). These findings are in contrast to the appearance of MRLC::GFP in fixed epithelial tissue of rotating and static egg chambers, where continuous filaments of Myo-II were seen and no large Myo-II dots were observed (S2 Fig and [28]).

Taken together, using high-speed confocal live imaging, we discovered that the MRLC of Myo-II displays a dot-like signal that is highly dynamic at the basal surface of the follicle epithelium and its speed of motion is independent of epithelial rotation during early and mid *Drosophila* oogenesis.

### The atypical cadherin Fat2 is required for planar symmetry breaking of Myo-II prior to the onset of epithelial rotation

Next, we investigated whether the small (~360nm) MRLC::GFP dots move in a specific direction with respect to the egg chamber anterior-posterior (AP) axis. To do this, we quantified MRLC::GFP movement directions, expressed as angles ranging from 0° to 360°, where 0° represented the anterior and 180° the posterior of egg chambers. These MRLC::GFP directions were then assigned to four 90° quadrants: Anterior (315°≤45°), Up (yellow, 45°≤135°), Posterior (135°≤225°) and Down (grey, 225°≤315°) (S1C Fig and Material and Methods). After quantification, we discovered that during rotation initiation (control and *fat2* mutant stage 1/2) MRLC:GFP showed a strong preference to move perpendicularly with respect to the AP axis of egg chambers (i.e. within Up and Down quadrants). This preference was only moderate during slow epithelial rotation (control stage 4) and strongly reinforced during fast (control stage 7) epithelial rotation (Fig 1B middle row and S3A Fig). In contrast, MRLC::GFP dots moved with no clear preferred direction in the epithelial plane in the static *fat2* mutant egg chambers of stage 7 (Fig 1B middle row). This data suggests that Fat2 is not required for Myo-II alignment during rotation initiation, but Fat2 is essential later for the maintenance of Myo-II alignment during epithelial rotation.

Similarly, labeling actin filaments with a LifeAct [29] molecule fused to GFP (LifeAct::GFP) showed a strong preference for LifeAct::GFP movement perpendicular to the AP axis of egg chambers during fast epithelial rotation (S1C and S3A Figs and S6 Movie). Indeed, the planar trend of MRLC::GFP and LifeAct::GFP signals, which were observed here using high-speed confocal imaging, corresponded to their signals in fixed controls and *fat2* mutant egg chambers during early and mid *Drosophila* oogenesis (S2 Fig).

Having established the planar trend of Myo-II movement perpendicular to the AP axis of rotating egg chambers, we next asked whether individual MRLC::GFP dots moved randomly along this planar trend. In order to do this, we plotted frequencies of MRLC::GFP dots within each defined quadrant in individual egg chambers. Strikingly, our data revealed that although MRLC::GFP dots moved in agreement with this planar trend, in individual egg chambers they only moved in one specific direction (i.e. either Up or Down) during rotation initiation (control stage 1/2), slow (control stage 4) and fast (control stage 7) epithelial rotation (Fig 1B last row). This Myo-II asymmetry was initially prominent during rotation initiation (control stage 1/2) and comparable to the asymmetry shown during fast epithelial rotation (control stage 7), but was less prominent during slow epithelial rotation (control stage 4). Further to this, no obvious asymmetry was detected in *fat2* mutant egg chambers of both stages 1/2 (rotation initiation) and stage 7 (no epithelial rotation) (Fig 1B last row). These results show that Fat2 is required for the planar symmetry breaking of Myo-II prior to the onset of epithelial rotation (during rotation initiation).

### Planar Myo-II preferentially moves against the direction of epithelial rotation

This preference for unidirectional Myo-II movement within a plane perpendicular to the AP axis, led us to speculate whether the particular direction of movement could relate to the eventual direction of epithelial rotation. It has been recently observed that *Drosophila* egg chambers can rotate in two possible directions (either clockwise or anti-clockwise) relative to their AP axis [8]. From our perspective, the clockwise direction corresponded to the direction of Myo-II movement within the Down quadrant, and anti-clockwise to the direction of Myo-II movement within the Up quadrant. Thus, we separately plotted the average percentage of MRLC::GFP dots moving within Up and Down quadrants and unified the direction of epithelial rotations in the Up direction for all analyzed rotating egg chambers. Notably, we detected that on average 59% and 77% of MRLC::GFP dots moved against epithelial rotation during slow and fast epithelial rotation, respectively (Fig 1C and S3B Fig). This was not true for the static *fat2* mutant egg chambers (stage 7), where no preferred planar direction of MRLC::GFP was identified in individual egg chambers (Fig 1C and Fig 1B). We also observed that actin molecules preferably moved (78% on average) against fast epithelial rotation, based on LifeAct-GFP, and this movement was comparable to the MRLC::GFP movement during fast epithelial rotation (S3B Fig, Fig 1C and S3 and S6 Movies). Importantly, based on our rotation initiation data, we observed that, although these egg chambers had not yet begun to rotate, MRLC::GFP dots strongly preferred to move (72% on average, comparable to control stage 7) towards one of the two possible directions (Up or Down) in individual egg chambers (Fig 1C and Material and Methods). In contrast, egg chambers lacking Fat2 did not show any clear preference in MRLC::GFP movement towards Up or Down during rotation initiation (Fig 1C).

In total, these data reveal that planar polarized Myo-II moves preferentially against epithelial rotation (henceforth called Myo-II retrograde movement). Notably, early planar symmetry breaking of Myo-II is Fat2-depenent and precedes the onset of epithelial rotation and, therefore, the decision whether egg chambers will rotate clockwise or anti-clockwise.

### The atypical cadherin Fat2 directs and reinforces Myo-II in individual follicle cells

**Fig 2:**
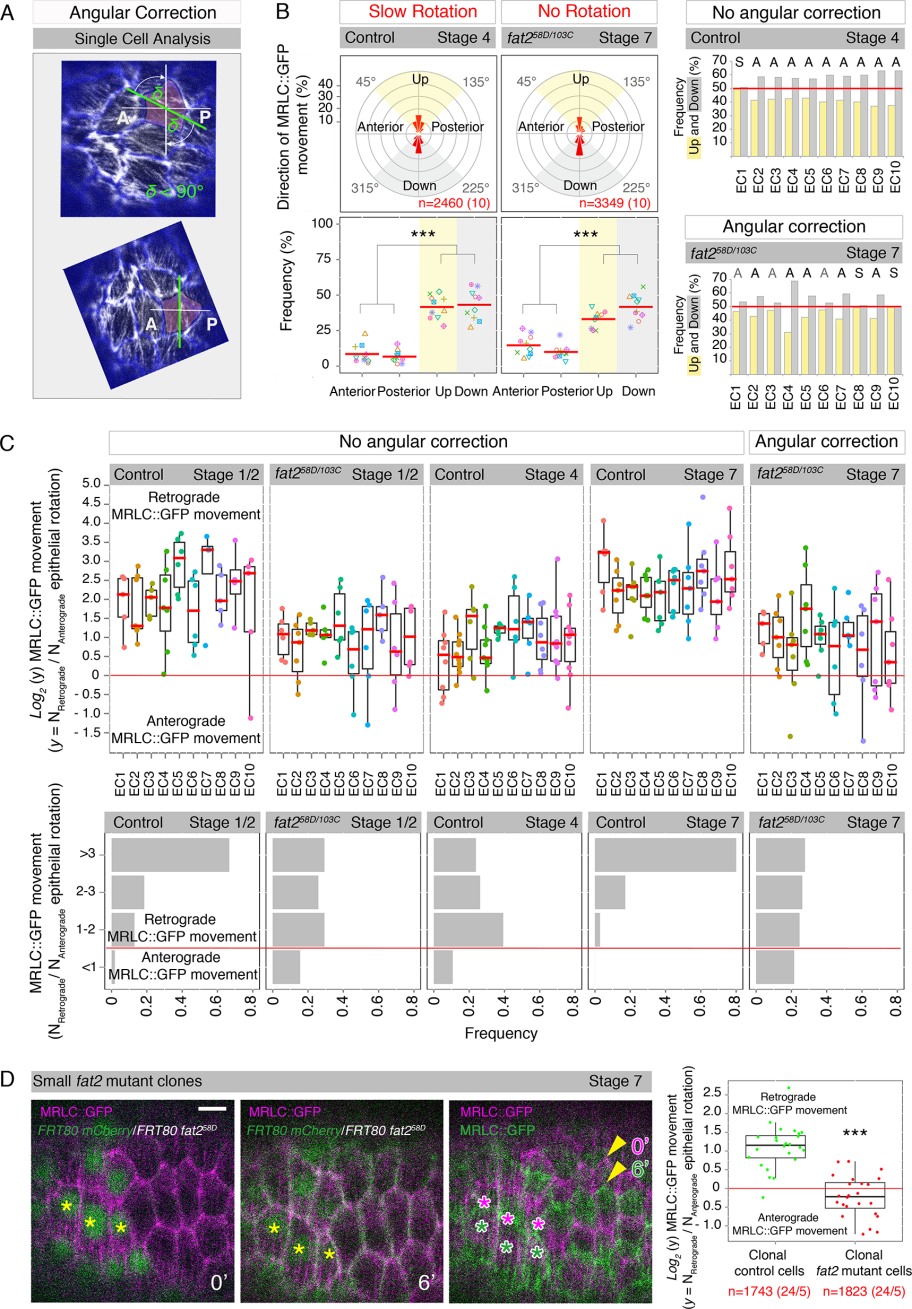
Fat2 locally both directs and reinforces Myo-II in individual follicle cells to promote epithelial rotation. **(A)** Angular correction approach (Material and Methods). Time-projected MRLC::GFP example of the angularly corrected image of *fat2* mutant follicle cells. A = anterior, P = posterior. **(B)** First row: Angular distribution and fractions of MRLC::GFP movements as described (Fig 1) for the angularly corrected (Material and Methods) stage 4 (slow epithelial rotation) and *fat2* mutant stage 7 (no epithelial rotation). Second row: Frequencies of MRLC::GFP movements for individual egg chambers after angular correction, in four quadrants (see description in Fig 1) with *P-*values < 0.001 (***) shown. Last panel: Frequencies of MRLC::GFP movement in Up and Down quadrants as a readout for Myo-II symmetry breaking in control stage 4 and the angularly corrected *fat2* mutant stage 7. A = asymmetry (black = strong, grey = weak), S = symmetry. **(C)** First row: Weighted ratios of MRLC::GFP moving against (retrograde) and with (anterograde) the direction of epithelial rotation, plotted on a *log2* scale. Dots represent individual follicle cells and colour individual egg chambers (EC). Box plots with median are shown. Symmetry border is at 0 (red line). Note that MRLC::GFP prefers to move mainly in a retrograde direction in individual follicle cells during rotation initiation (control stage 1/2) and fast epithelial rotation (control stage 7) in contrast to no epithelial rotation (stage 1/2 and stage 7) and slow epithelial rotation (stage 4) where a few cells also show anterograde MRLC::GFP movement. Second row: Frequencies of binned MRLC::GFP ratios over analyzed egg chambers and types of epithelial rotation are shown. **(D)** Ratios of MRLC::GFP, moving against (retrograde) and with (anterograde) the direction of epithelial rotation, analyzed in individual follicle cells of small *fat2* mutant clones (lack of green nuclei, Material and Methods) and their control neighbours (green nuclei) are plotted on a *log_2_* scale. The number of analyzed MRLC::GFP signals is indicated in red (number of analyzed follicle cells/egg chambers is shown in brackets). Coloured stars in images indicate the direction of epithelial rotation. Time and *P-*value < 0.001 (***) are shown. Scale bar = 5μm. Anterior is on the left.

Next, we sought to uncover how Fat2 breaks Myo-II symmetry in the follicle epithelium during rotation initiation and how it regulates Myo-II retrograde movement during epithelial rotation. We have shown here that Fat2 does not play a role in the planar alignment of Myo-II during rotation initiation (Fig 1B middle row). Therefore, we assumed that Fat2 must use a different unrelated mechanism, in addition to the planar alignment of Myo-II movement, to break Myo-II symmetry in the follicle epithelium of young egg chambers during rotation initiation. To support this hypothesis, we took advantage of our finding that Fat2 regulates the planar alignment of Myo-II perpendicular to the AP axis of rotating egg chambers (based on live imaging and fixed tissue, Fig 1 and S2 Fig), but does not seem to have an impact on local Myo-II alignment within follicle cells (S2 Fig). To this end, we hypothesized that artificial planar alignment of individual *fat2* mutant follicle cells perpendicularly to the AP axis of egg chambers should not in principle be sufficient to break the symmetry in the follicle epithelium. To simulate such a situation, we developed a computational angular correction approach, in which we assumed that such epithelial remodeling (either by dissolving/reestablishment of adherens junctions or cell intrinsic regulation of the actomyosin cytoskeleton) happens in the wild-type situation with minimal movement of the respective components (i.e. cells or cytoskeleton would rotate 45 rather than 135 degrees to align). To mimic this, individual *fat2* mutant follicle cells were angularly corrected for their predominant MRLC::GFP direction by the smallest possible angle (i.e. < 90°) to reach perpendicular alignment to the AP axis of individual egg chambers (Fig 2A and Material and Methods). When angular correction was applied, we observed that although MRLC::GFP dots had moved perpendicular to the AP axis in the static *fat2* mutant follicle epithelia (stage 7), it was only to the extent of the angularly corrected control stage 4 (slow epithelial rotation) Fig 2B. This is in contrast to control stage 7, which displayed a strong planar trend for MRLC::GFP movement (fast epithelial rotation, Fig 1B middle row), indicating that either Fat2 or epithelial rotation are responsible for reinforcement of planar Myo-II alignment during fast epithelial rotation. Notably, out of ten analyzed independent egg chambers only five clearly broke planar Myo-II symmetry (at least comparable to the control stage 4), three displayed very weak asymmetry and two remained symmetrical (Fig 2B). These findings indicate that proper alignment of Myo-II movement perpendicular to the AP axis of rotating egg chambers is not sufficient in itself to break the planar symmetry of Myo-II during fast epithelial rotation. Therefore, it seems that to break Myo-II symmetry in the follicle epithelium prior to and during epithelial rotation, an unknown Fat2-dependent mechanism is necessary.

Based on this finding, we hypothesized that Fat2 could potentially regulate Myo-II dynamics directly at the intracellular level. To support this hypothesis, we first analyzed the behaviour of MRLC::GFP dots in individual follicle cells of control and *fat2* mutant egg chambers during rotation initiation, slow epithelial rotation, and fast epithelial rotation (Fig 2C). To be able to compare between these situations, we plotted the direction of MRLC::GFP dots as the weighted ratio of those moving against epithelial rotation (retrograde MRLC::GFP dots) versus those moving with it (anterograde MRLC::GFP dots) (Fig 2C, Material and Methods). In the case of static egg chambers (*fat2* mutant egg chambers of stages 1/2 and 7), we plotted the weighted ratio of MRLC::GFP dots with strong preference towards one of the two possible directions (Up or Down) to those in the opposite direction (Material and Methods). Only *fat2* mutant egg chambers of stage 7 were angularly corrected, in order to compare with the other situations. Interestingly, this analysis of Myo-II behaviour at the intracellular level revealed that individual follicle cells of an egg chamber displayed different MRLC::GFP ratios (Fig 2C). However, when considering all analyzed egg chambers of a given situation, MRLC::GFP ratios tended to show significantly higher ratios (>3, on *log*_*2*_ scale > 1.6) for rotation initiation (control stage 1/2) and fast epithelial rotation (control stage 7). This was in contrast to slow epithelial rotation (control stage 4) with a ratio preference between 1–2 (on *log*_*2*_ scale 0–1) and static *fat2* mutant egg chambers (stage 1/2 and stage 7), which displayed rather equal distribution of MRLC::GFP ratios when binned to categories (<1, 1–2, 2–3 and >3, Fig 2C and S4A Fig). Notably, we identified one or more follicle cells of individual egg chambers with symmetric MRLC::GFP movement or with anterograde direction (opposite to the overall preferred direction) during rotation initiation, slow epithelial rotation, and in static *fat2* mutant egg chambers (Fig 2C and S4A Fig). This was never the case during fast epithelial rotation when all egg chambers displayed only follicle cells with strong retrograde MRLC::GFP movement (Fig 2C). Similarly, we observed the same, exclusively retrograde movement with LifeAct::GFP in individual follicle cells during fast epithelial rotation (S4B and S4C Fig), indicating that MRLC::GFP movement reliably reflects LifeAct::GFP behaviour.

In summary, the reason why 50% of static *fat2* mutant egg chambers (stage 7) did not clearly break planar symmetry of Myo-II (Fig 2B) is that they contained one or more follicle cells with strong MRLC::GFP movement in the opposite direction to the overall preferred one within the whole follicle epithelium (Fig 2C). As a similar situation was observed in *fat2* mutant egg chambers during rotation initiation, we conclude that the Fat2 intracellular function is likely to guarantee that Myo-II moves in the same preferred direction in all follicle cells within the follicle epithelium to break Myo-II symmetry. This finding shows that although neighbouring follicle cells in static *fat2* mutant egg chambers can sense the planar alignment of actin filaments between their neighbours (based on fixed tissues in the 0°-180° range [30]), when we use high speed live imaging and quantify in the 0°-360° range, it can be clearly seen that neighbouring follicle cells actually cannot sense the direction of Myo-II among each other (often found in opposing direction Fig 2C).

To prove that Fat2 specifically regulates Myo-II behaviour at the intracellular level, we generated mosaic egg chambers with substantially small *fat2* mutant clones (Material and Methods), which resulted in rotating egg chambers with a speed comparable to control egg chambers of similar stage (S5A Fig and S7 Movie). Using high-speed confocal live imaging, we then analyzed the MRLC::GFP behaviour at the basal surface of these *fat2*^*58D*^ mutant follicle cells and compared it to the MRLC::GFP behaviour of their direct control neighbouring follicle cells (Fig 2D and S7 Movie). Similarly as shown in Fig 2C, we plotted, on a *log*_*2*_ scale, MRLC::GFP movement as a ratio of MRLC::GFP dots moving against (retrograde) epithelial rotation versus those moving with (anterograde) epithelial rotation. This approach revealed that clonal control follicle cells contained Myo-II that displayed a significant preference for retrograde movement, whereas clonal *fat2* mutant follicle cells showed no preferred direction with respect to epithelial rotation (Fig 2D). In addition, follicle cells in the *fat2* mutant small clones did not lose their intracellular Myo-II alignment perpendicular to the AP axis of mosaic egg chambers (S4D Fig).

Thus, our data provide evidence that it is not epithelial rotation *per se* that impacts Myo-II asymmetries (both direction and magnitude), but instead, it is a specific function of Fat2 that is required to direct Myo-II movement against epithelial rotation and reinforce this retrograde Myo-II movement at the intracellular level of individual follicle cells upon the onset of fast epithelial rotation. Moreover, we also show that follicle cells do not need Fat2 to sense the planar Myo-II alignment of their neighbours in mosaic egg chambers with small *fat2* mutant clones.

### Epithelial rotation both drives and directs the elongation of follicle cells

**Fig 3:**
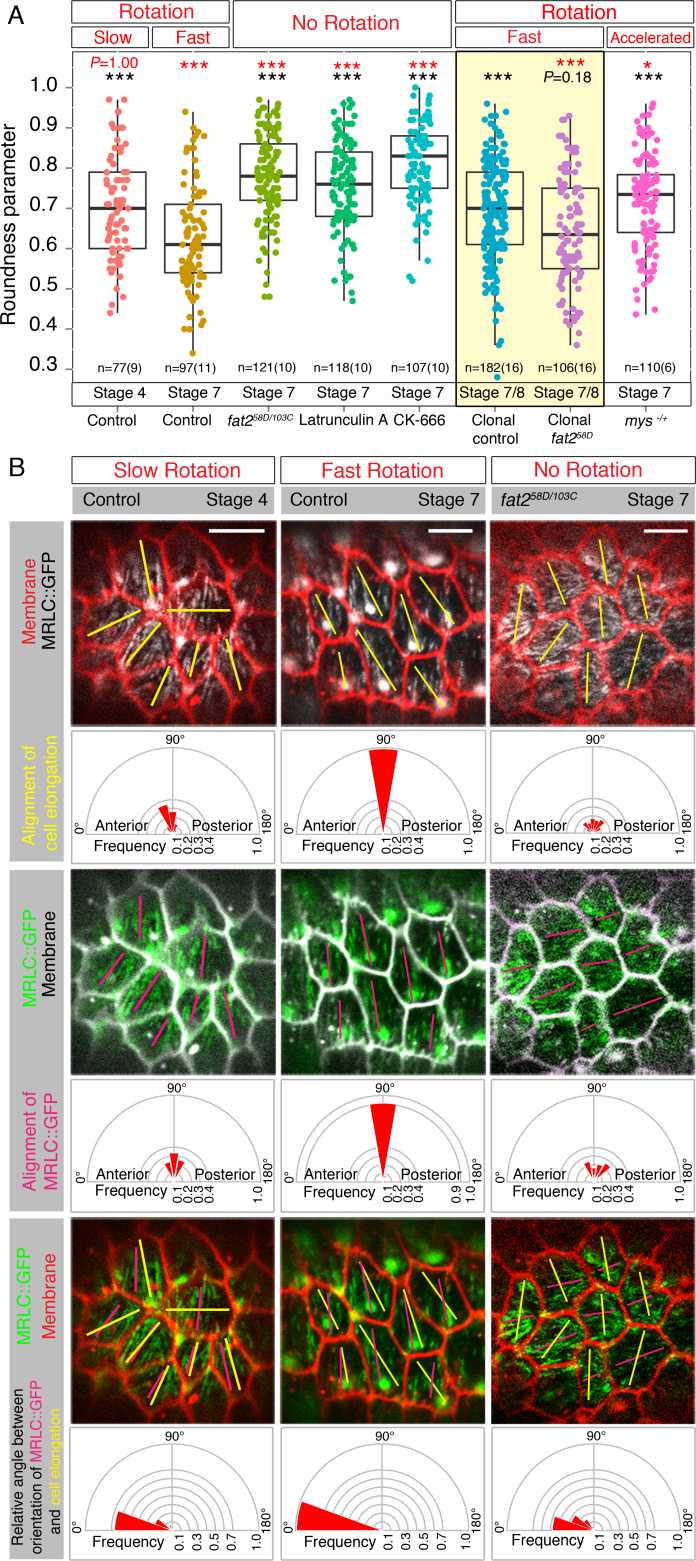
Epithelial rotation is required for the directed elongation of follicle cells. **(A)** Comparison of roundness parameter (Material and Methods) of follicle cells with different migratory speed: slow migrating follicle cells (control stage 4), fast migrating follicle cells (control stage 7), follicle cells in the static follicle epithelium (*fat2* mutant follicle cells), follicle cells depleted for actin, Latrunculin A and depleted for Arp2/3 complex, CK-666, migrating *fat2* mutant follicle cells in small clones and their migrating control neighbours (in yellow panel) and follicle cells with accelerated migration (*mys* heterozygous mutants). *** = *P*<0.001, * = *P*<0.05. Note that the roundness parameter of analyzed categories was statistically compared to the one with no indicated stars. Black *P*-values indicate how the roundness parameter differs from the control stage 7 (fast epithelial rotation). Red *P*-values show how the roundness parameter differs from the clonal control stage 7/8 (fast epithelial rotation in the *fat2* mutant mosaic eggs chambers). **(B)** Angular distribution of alignment of cell elongation and of MRLC::GFP movement expressed as frequencies in 20°-bin-rose diagrams are shown for slow (control stage 4), fast (control stage 7) and no (*fat2* mutant of stage 7) epithelial rotation. Yellow bars show the direction of follicle cell elongation and berry-coloured lines the direction of local Myo-II in the range of 0°-180°. Scale bars = 5μm. Anterior is on the left.

Next, we wished to understand the role that epithelial rotation plays in the follicle epithelium. Epithelial rotation has been clearly linked to the PCP of the basement membrane and actin filaments, which is necessary for proper elongation of egg chambers along their AP axis [17, 18]. In addition, integrin-based adhesions to the ECM can modulate the speed of epithelial rotation and impact the shape/stretching of follicle cells [17]. Therefore, we wondered whether epithelial rotation could define the shape of follicle cells. To investigate this, we measured the roundness parameter of the basal surface of follicle cells (Material and Methods) and found that follicle cells are on average significantly more elongated during fast epithelial rotation and less elongated during slow epithelial rotation. This was in contrast to the round follicle cells observed in static *fat2* mutant egg chambers (Fig 3A). We also pharmacologically perturbed epithelial rotation by using the actin-depleting drug, Latrunculin A, and the Arp2/3 complex-depleting drug, CK-666, which have been shown to stop epithelial rotation [18, 20]. Both these pharmacological experiments resulted in static follicle cells and the familiar round shape observed in *fat2* mutant follicle cells (Fig 3A and S5A Fig, S8 and S9 Movies), indicating that epithelial rotation is required for the elongation of follicle cells. To distinguish whether the round shape phenotype of *fat2* mutant follicle cells is a result of the absence of epithelial rotation in analyzed *fat2* mutant egg chambers or an actual Fat2 specific function that is required for cell elongation, we analyzed the follicle cells of mosaic egg chambers that contained small *fat2*^*58D*^ mutant clones and displayed similar speed to control egg chambers of a comparable stage (S5A Fig, Material and Methods). To our surprise, clonal control cells elongated to the same extent as slowly migrating follicle cells (control stage 4), but were significantly less elongated than neighbouring clonal *fat2* mutant follicle cells. These *fat2* mutant follicle cells displayed the same elongation as the fast migrating follicle cells of control stage 7 egg chambers (Fig 3A) and indicated that Fat2 could be required to provide follicle cells with resistance against cell stretching at their basal side likely via Myo-II regulation (Fig 2D). We further confirmed that weakening the attachment to the ECM via decreased integrin level led to accelerated speed of epithelial rotation (S5A Fig, [17]) and resulted in similar cell elongation to that found in clonal control follicle cells (stage 7/8) and slow migrating follicle cells in egg chambers of control stage 4 (Fig 3A). In addition, this clonal analysis suggested that *fat2* mutant cells, with impaired resistance to cell stretching, influence the cell elongation of their neighbouring controls.

As we observed that follicle cells were elongated along the direction of epithelial rotation in fast rotating egg chambers (e.g. S3 Movie), we wished to know whether epithelial rotation could define the direction of elongation of follicle cells. We therefore quantified the alignment of cell elongation, expressed as the angular direction, in 20 degree bins, through a range of 0°-180° during slow, fast and no epithelial rotation. We found that follicle cells elongated mainly perpendicularly to the AP axis of egg chambers during fast epithelial rotation and that this was weaker during slow epithelial rotation (Fig 3B). To understand how the elongation of follicle cells relates to the planar alignment of Myo-II in individual follicle cells, we calculated the relative angle between the planar alignment of follicle cell elongation and the MRLC::GFP pattern (Material and Methods). We revealed that the direction of MRLC::GFP movements were in the plane of follicle cell elongation 65% of the time during slow epithelial rotation and this increased to 100% during fast epithelial rotation (Fig 3B). The planar alignment of the MRLC::GFP pattern preceded that of follicle cell elongation during slow epithelial rotation, indicating that epithelial movement prefigures the elongation of follicle cells. In contrast, *fat2* mutant follicle cells displayed random planar alignment in their elongation, MRLC::GFP pattern, and relative angle.

Altogether, this data shows that epithelial rotation (clockwise or anti-clockwise) is required for the proper elongation of follicle cells and the planar alignment of their elongation in the direction of epithelial rotation (henceforth called directed elongation) perpendicular to the AP axis of rotating egg chambers.

### Epithelial rotation suppresses anisotropic and premature Myo-II pulses to protect epithelial tissue from cell deformations

**Fig 4:**
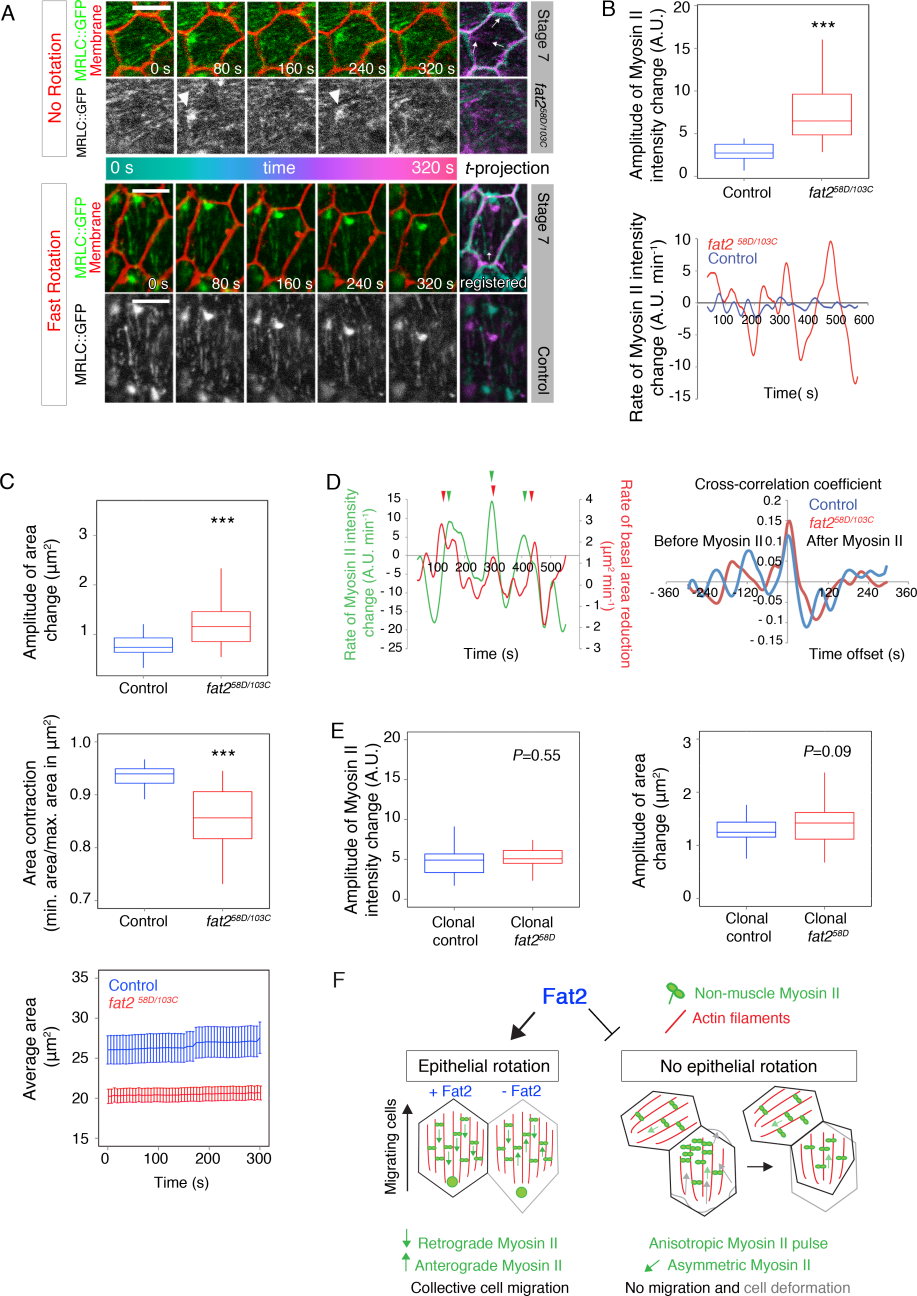
Epithelial rotation suppresses premature Myo-II anisotropic pulses and cell deformations. **(A)** Unequal MRLC::GFP intensity increments (Myo-II anisotropic pulse) and membrane deformations are shown in *fat2* mutant follicle cells (stage 7). Arrowheads indicate an individual MRLC::GFP intensity increase (a pulse). Note that MRLC::GFP pulses were not observed in control stage 7 (data for one representative cell is shown). Scale bars = 5μm. Anterior is on the left. **(B)** Amplitude of Myosin-II intensity change and a single cell example of control and *fat2* mutants plotting the rate of Myo-II intensity change, showing significant Myo-II pulses in *fat2* mutant follicle cells. **(C)** Amplitude of area changes were significantly higher in *fat2* mutant follicle cells than in controls. Correspondingly, area contraction [ratio of the minimal area (contraction) to the maximal area (relaxation)] was significantly weaker in *fat2* mutant follicle cells. Average area of analyzed follicle cells, which was calculated for *fat2* mutant follicle cells and their controls over time (300s) with S.E.M. is shown. **(D)** Rate of MRLC::GFP intensity change (green) and rate of the basal area reduction (red) in one representative cell temporally correlates. Peaks are marked with arrowheads of corresponding colour. Cross-correlation coefficient (average of all correlation coefficients with different time offsets) between rate of MRLC::GFP intensity and rate of follicle cell area reduction (red line) is shown. *R* = 0.15 with peak maxima at +6s, indicating that MRLC::GFP precedes the basal area reduction. Blue line shows the average time-dependent correlation of the control between rates of MRLC::GFP intensity change and basal area reduction. *R* = 0.11 with a maximum peak at 0, suggesting that MRLC::GFP and basal area reduction are almost simultaneous. **(E)** Comparison of amplitude of Myo-II intensity change and of area change did not show significant difference between clonal *fat2* mutant cells (of small clones) and their control neighbours. **(F)** Fat2 suppresses unwanted Myo-II anisotropic pulses/cell deformations via facilitating epithelial rotation. Based on data from Figs 2D and 3A, Fat2 presence is also required for proper Myo-II dynamics and cell elongation control during the migratory phase. *** = *P*<0.001 and *P* values are shown. Follicle cells of stage 7 were used for both, *fat2* mutant (n = 56, 7 independent egg chambers) and control (n = 28, 5 independent egg chambers) analysis (B, C, D). Follicle cells of stage 7/8 were used for clonal analysis (n = 24, 5 independent egg chambers in E).

We next asked what impact epithelial rotation has on the Myo-II behaviour in the epithelial tissue of egg chambers. When we analyzed individual follicle cells within a *fat2* mutant follicle epithelium, besides loss of planar Myo-II alignment and weak Myo-II asymmetries, we also observed spatially unequal (i.e. anisotropic) MRLC::GFP pulses (Fig 4A–4C and S5B and S5C Fig) along with the constant remodeling and deformation of cellular membranes (Fig 4C and S5B Fig). Membrane deformation resulted in significant basal area contractions in *fat2* mutant follicle cells compared to the corresponding control (Fig 4C). However, average area stayed unchanged, indicating that these area contractions were asynchronous among neighbouring *fat2* mutant follicle cells (Fig 4C). We observed that the reduction in basal area followed ~6s after the increase of MRLC::GFP, based on our calculated cross-correlation coefficient (Fig 4D and Material and Methods). However, this cross-correlation coefficient is weak, indicating that alternating anisotropic Myo-II pulses in the follicle epithelium are influenced by external forces generated in neighbouring follicle cells, which in turn have an impact on the shape of neighbouring membranes. In contrast, this pulsating behaviour was missing in the corresponding control (stage 7) during fast epithelial rotation (Fig 4A and 4B and S5C Fig). Importantly, our clonal analysis of rotating mosaic egg chambers that contained small *fat2* mutant clones (Fig 2D, Fig 3A, Material and Methods) showed no significant Myo-II pulses and no area change in clonal *fat2* mutant follicle cells (Fig 4E). Although not significant, the area change of these cells appeared more frequent than that of control follicle cells. Thus, our data provide evidence that epithelial rotation suppresses anisotropic Myo-II pulses and cellular membrane contractions/relaxations to prevent deformations of epithelial tissue (Fig 4F). This data also supports our previous observation where we saw basally deformed follicle cells in the fixed follicle epithelium of *fat2* mutant egg chambers [25]. In addition, our observation shows that *fat2* mutant follicle cells likely suffer from impaired cell retraction, as recently observed by others [28].

## Discussion

In single animal cells, it is essential to break symmetry to establish intracellular polarization, which in turn defines the direction of cell locomotion. The motility of a cell can be provided by protrusive forces, as a result of actin and MT polymerization, and/or by tensile forces from myosin motors that contract cross-linked actin filaments [31]. Myosin motors that bind actin filaments generate contractile forces, resulting in actin deformations and allow actomyosin networks to behave like a flowing viscoelastic fluid [32]. Actomyosin flows can be classified as transient, sustained, and oscillatory based on their character [31]. Transient actomyosin retrograde flow can be observed, for example, in the one and four-cell *C*. *elegans* embryo [14], where the actomyosin network provides active torque that generates force, which leads to symmetry breaking along the embryonic axes.

Sustained actomyosin flows are often of a retrograde (i.e. from the front to the rear of the migrating cell) character and can be divided into two groups dependent on their style of cell locomotion. Firstly, this locomotion can be a result of high cortical contractility [33], where no protrusions and no adhesion to a substrate is used, allowing cell mass to be pushed forward in front of the cell (e.g. swimming cells). Secondly, an alternative type of locomotion observed classically in fish keratocytes [34] or neuronal growth cones [35] can be a result of low cortical contractility, when protrusions and substrate/ECM adhesions are used to pull the cell forward with retraction of the rear end [31]. Even though this type of actomyosin retrograde flow is typically observed in single motile cells, it has been also identified in the yolk cell of the zebrafish embryo during the time when the enveloping cell layer is spreading over the yolk cell by using a strong actomyosin contractile ring that combines contractile forces and flow-friction mechanisms based on actomyosin retrograde flow [36].

Here, we identified another, up to now unknown, polarized and dynamic actomyosin network that shows preferred actomyosin retrograde movement/flow at the basal surface of protrusive and ECM-adhesive follicle cells in the follicle epithelium of acinar-like *Drosophila* egg chambers. Interestingly, we found that the speed of Myo-II in *Drosophila* egg chambers corresponds very well to the anterograde flow of actomyosin observed during zebrafish gastrulation [36]. Given the fact that this preferred actomyosin retrograde flow covers the whole basal surface of follicle cells, we propose that follicle cells use directed basal actomyosin tensile contractility on their circumference as the main candidate force-generating mechanism, combined with supportive protrusive force, via actin-rich protrusions[18, 23], to actively move the whole mass of follicle cells in a collective manner. In light of our work, the previously proposed actomyosin-based ‘molecular corset’ that has been suggested to restrict the elongation of egg chambers [19, 37] along their dorso-ventral axis, turns out to in fact be a highly dynamic and polarized actomyosin network that drives epithelial rotation in order to protect follicle cells from their deformations and only later serves as a restrictive mechanism that limits the oocyte expansion via polarized Myo-II pulses [38].

The third and final classification of actomyosin flows relates to oscillatory flows. These can be found in animal tissues, where the actomyosin network often oscillates and can change its direction. Such flows can be observed in junctional remodeling during *Drosophila* embryonic germband extension [39], or neuroblast ingression, [40] and have been referred to as actomyosin pulses, for example, during dorsal closure and formation of the ventral furrow in *Drosophila* embryos [41, 42]. Similar Myo-II oscillations were also observed at the cellular surface (basal side) of follicle cells during mid-late *Drosophila* oogenesis [38]. Although all these examples of actomyosin/Myo-II oscillations/pulses are tightly linked to proper embryogenesis/tissue morphogenesis, our work reveals that, at least in the *Drosophila* follicle epithelium, Myo-II pulses are not *per se* a guarantee of proper morphological process. This is evident because *fat2* mutant egg chambers display Myo-II pulses, but do not properly elongate (i.e. they remain round) in late *Drosophila* oogenesis. We hypothesize that these observed non-physiological Myo-II pulses may be a consequence of a tug-of-war between neighbouring cells or the absence of communication between follicle cells and the ECM. The first is based on the existence of weak Myo-II asymmetries in *fat2* mutant follicle cells that likely generate directed force. These forces are often in opposing directions (Fig 2C) and may result in Myo-II pulses as neighbouring cells fight as to which direction to collectively move. The second explanation is based on the possibility that communication with the ECM is defective or impaired. We speculate that actin-rich protrusions, which have previously been linked to epithelial rotation [18, 23, 28], may have a mechanosensitive function and therefore are able to sense the behaviour of neighbouring cells, or even those at a distance through the ECM. In the absence of such ECM-cell communication the generated force needs to be released to the ECM if not utilized for the migration of follicle cells, resulting in the observed Myo-II pulses.

Mechanical forces are essential for cell rearrangements and tissue morphogenesis during embryogenesis. For example, it has been shown recently that the movement of neighbouring tissues can establish friction forces at their interface, and these forces can be critical for forming correctly the shape of a zebrafish embryo [43]. Here we show that the level of attachment to the ECM is one determinant that defines to what extent follicle cells elongate. Thus, a similar friction-based mechanism is likely involved in the follicle epithelium, resulting in the directed elongation of follicle cells. In fact, the force generated by the collective movement of follicle cells could be plausibly transmitted through the ECM, and provide long-range signaling as previously suggested [31, 44, 45]. This view supports the recent finding that force can be transmitted between cells and within tissue, and is a critical factor for the organization of the actomyosin network during dorsal closure in the *Drosophila* embryo [46, 47] and in the *Drosophila* wing [48]. The essential function of the ECM in collective cell migration and egg chamber morphogenesis has been recently confirmed [49, 50].

Little is known about the mechanistic control of symmetry breaking in epithelial animal tissues. It has been only recently discovered that atypical cadherins can act via force generating molecules, such as the unconventional myosins, Dachs and MyoID [51, 52]. Our data show that another atypical cadherin, namely Fat2, controls symmetry breaking by regulation of cellular mechanics via conventional non-muscle Myo-II. We have discovered that Fat2 is required: (i) to unify Myo-II asymmetries in one direction in individual follicle cells in order to break planar symmetry of Myo-II prior to the onset of epithelial rotation; (ii) to guarantee the presence of unidirectional actomyosin contractility by correcting opposing Myo-II symmetries/asymmetries; (iii) to guarantee proper Myo-II alignment in individual follicle cells perpendicular to the AP axis of egg chambers during slow epithelial rotation and (iv) to reinforce retrograde Myo-II asymmetries and planar Myo-II alignment during fast epithelial rotation. Only then can unidirectional actomyosin contractility, at the central basal surface of follicle cells, together with actin-rich protrusions, at the front of the basal side of follicle cells, drive and likely predict the direction of epithelial rotation and subsequent directed cell elongation. As Fat2 is similarly required for the symmetry breaking of MTs prior to the onset of epithelial rotation [20, 24], it will be interesting to find out in the future, what interdependencies Myo-II and MTs display. It will also be interesting to identify why Fat2 breaks the symmetry of both these cytoskeletal components at the time of rotation initiation in *Drosophila* egg chambers in order to initiate the rotational movement of this acinar-like invertebrate organ. Thus, it appears that the atypical cadherin subfamily likely developed a prominent function to shape tissues in two ways, dependent on tissue character. Firstly, in migratory tissue via retrograde Myo-II movement and an ECM-dependent mechanism (Fat2-Myo-II in *Drosophila* egg chambers in this work) and, secondly, in moving non-ECM-migratory tissue via intercalations. For example, Dachsous-MyoID in the *Drosophila* hindgut [52] and Fat-Dachsous-Dachs in the *Drosophila* wing [53]. However, it remains unclear exactly which signal instructs cadherins to initiate the breaking of symmetry in these epithelial tissues and it needs to be addressed in the future whether the signal is of biochemical or physical (cadherins are force-inducible as observed in the C-cadherin/keratin complex in *Xenopus* [54]) character.

Fat2 close homologs, namely Fat1-3, exist in vertebrates [55] and have been implicated in cancer [56] and autism [57]. Surprisingly, the intracellular domain of mouse Fat3, whose cell-autonomous function has been recently linked to intracellular actin cytoskeleton organization via the Ena/VASP complex in directed cell migration and trailing edge retraction of amacrine cell in the mouse retina [58], resembles *Drosophila* Fat2 function in collective cell migration [18, 20], directed cell elongation (this work) and retraction of follicle cells [28] during *Drosophila* oogenesis. Thus, it is likely that a similar conserved mechanism is used to move and sculpt tissues of tubular/acinar organs in vertebrates.

## Materials and methods

### Fly stocks and genetics

The *Drosophila* MRLC (myosin regulatory light chain of the non-muscle conventional Myosin II), encoded by *spaghetti-squash* (*sqh*), was visualized by MRLC fused with eGFP [27] under the *sqh* promoter in a null *sqh*^*AX3*^ or *sqh*^*AX3*^/*sqh*^*AX3*^*;; fat2*^*58D*^*/fat2*^*103C*^ mutant background to avoid competition with the endogenous protein. The following stocks and genotypes were used: *sqh*^*AX3*^*/ sqh*^*AX3*^*; sqh-MRLC*::*GFP/ sqh-MRLC*::*GFP* (on the II. chromosome) and *sqh*^*AX3*^*/sqh*^*AX3*^*;sqh-MRLC*::*GFP/ sqh-MRLC*::*GFP*; *fat2*^*58D*^*/fat2*^*103C*^ were used in all figures except for S2 Fig, where the *sqh-MRLC*::*GFP/ sqh-MRLC*::*GFP*; *fat2*^*58D*^*/ fat2*^*103C*^ line was used for *fat2* mutant egg chambers. For accelerated epithelial rotation, we used *mys*^*XB87*^ *FRT18*/*FM7*.

To visualize actin filaments by time-lapse life imaging, we used *Act5C-Gal4* (BL4414, on II. chromosome)>*UAS-LifeAct*::*GFP* (on II. chromosome) shown in S6 Movie. To label cell membranes we used CellMask^TM^ Deep Red (Invitrogen).

### Clonal analysis

Mosaic egg chambers were generated using the FRT-Flp system [59] when 1–2 days old adult flies were exposed to a 38°C heat-shock bath for half an hour (2 times a day) on three successive days. Ovaries were dissected 5–7 days after the last heat-shock. The analyzed genotypes were: *y w hsp-flp/+*; *sqh*-*MRLC*::*GFP*/*sqh*-*MRLC*::*GFP; FRT80B*, *fat2*^*58D*^*/FRT80B*, *ubi-mCherry* (Fig 2D, Fig 3A, Fig 4E and S5 Fig).

### Time lapse imaging

Egg chambers were cultured and live imaging performed as described [20]. Notably, we used no agarose for egg chamber embedding and no cover slip to avoid any potential artificial forces. An inverted LSM 700 Zeiss confocal microscope was used with 63x/1.45 water immersion lens. Time-lapse movies were taken with an interval of 6s for 300s-600s.

### Fixation and immunohistochemistry

Adult fly ovaries were dissected in 1xPBS and fixed with 4% *p*-formaldehyde for 20 minutes. Immunostaining followed standard protocols. We used a polyclonal GFP tag antibody conjugated with Alexa Fluor 488 (Molecular Probes) in dilution of 1:100 and rhodamine-phalloidin in dilution of 1:200. Images were acquired on an inverted LSM700 Zeiss confocal microscope with a 63x/1.45 oil immersion lens.

### Drug treatments

To inhibit polymerization of actin filaments, Latrunculin A (10 μM in 1% DMSO, Enzo Life Sciences) was used for ca. 10mins before direct imaging. To deplete actin protrusions, we used an Arp2/3 inhibitor (CK-666, 250 μM, Sigma) for ca. 1h as described [18].

### Image processing and data analysis

Measurement of the direction of Myo-II movement, size and velocity, epithelial rotation velocity, angular correction, actomyosin directionality, cell shape, Myo-II intensity and PCC are described in S1 Text.

### Data plotting and statistics

Rose diagrams, quadrant plots, histograms, bar and box plots were created in R studio http://www.rstudio.com, using various packages [60–63]. Error bars represent standard error of the mean (S. E. M.). The Student double-sided t-test was used as indicated.

## Supporting information

S1 Text Supporting materials and methods. (DOCX)

S1 Fig The PCC role in symmetry breaking, the Fat2 role in rotational speed during early and mid-oogenesis and analysis of actomyosin movement. **(A)** Planar cell chirality (PCC) does not seem to significantly contribute to symmetry breaking during rotation initiation (stage 1/2), slow (stage 4) and fast (stage 7) rotating follicle epithelium. Number of cell membranes n = 110, n = 261 and n = 319 over 10 independent egg chambers were analyzed for rotation initiation, slow and fast epithelial rotation, respectively. S.E.M. is shown along with P values. ACW = anti-clockwise and CW = clockwise epithelial rotation, when observed from the anterior tip of an egg chamber. **(B)** Rotational speed (μm/min) during rotation initiation (stage 1/2), slow (stage 4), fast (stage 7) and not rotating (*fat2* mutant) egg chambers (stage 1/2, stage 7) is shown. **(C)** Intracellular MRLC::GFP individual dot-like and LifeAct::GFP signals (example indicated with yellow circle) were analyzed at the basal surface of follicle cells. Direction of their movement (based on time-projected images, note the colour-coded *t*-projection, the green arrow shows the direction corresponding to almost 270° selected in yellow circle) was expressed as angle in the range of 0°-360°. Examples of stage 7/8 (fast epithelial rotation) are shown. Kymographs showing retrograde movement of MRLC::GFP and LifeAct::GFP signals (white arrows). Scale bars = 5μm. Anterior is on the left.
(TIF)

S2 Fig Planar alignment of Myo-II and actin filaments in the fixed follicle epithelium. **(A)** Planar alignment of MRLC::GFP (green) and actin filaments (red) to the AP axis (0°-180°) at the basal side of the fixed *Drosophila* germarium, showing strong perpendicular alignment to the AP axis during rotation initiation, which is temporarily decreased when the egg chamber buds from the germarium (stage 2) during early oogenesis (represented by stage 4) and reaches its proper perpendicular alignment at the time of fast epithelial rotation (represented by stage 7), which is still present at stage 9 when egg chambers cease their epithelial rotation. In *fat2* mutant fixed egg chambers, the MRLC::GFP planar polarized pattern was globally disturbed and reflects the direction of actin filaments at stage 7 and stage 9. White boxes show the magnification of a representative follicle cell of a particular stage, which display local MRLC::GFP signal localization. Note that MRLC::GFP displays irregular signal distribution in *fat2* mutant egg chambers (stage 7 and 9) compared to corresponding controls with local MRLC::GFP asymmetric distribution during oogenesis with the epithelial rotation (stage 7). **(B)** Histograms represent frequency distribution of angles of MRLC::GFP movement and actin filaments (F-actin) measured between 0° and 180°. Anterior (0°) is on the left, posterior (180°) is on the right. S.E.M. is shown. Scale bars = 5μm, except of stage 9 where scale bar = 10μm.
(TIF)

S3 Fig Symmetry breaking of actomyosin and its preferred movement relatively to epithelial rotation. **(A)** First row: Angular distribution of MRLC::GFP movement expressed as frequencies plotted in 20 degree-bin rose diagrams during fast epithelial rotation (stage 6 and stage 8) is compared to LifeAct::GFP signals (stage 8). Second row: Frequencies of MRLC::GFP and LifeAct::GFP movement in four 90 degree quadrants are plotted, showing that the significant (*** = *P*<0.001) majority of MRLC::GFP moves within Up (yellow) and Down (grey) quadrants. The number of analyzed MRLC::GFP and LifeAct::GFP signals are indicated in red in the lower right with the number of independently analyzed egg chambers (in brackets). **(B)** Frequencies of MRLC::GFP movement in Up (yellow, 45°≤135°) and Down (grey, 225°≤315°) quadrants during fast (stage 6 and 8) epithelial rotation that was unified to the Up direction of epithelial rotation. Similarly, Up and Down frequencies of LifeAct::GFP movement are shown for fast epithelial rotation (stage 8) that was unified to the direction Up. Note that strong retrograde movement of LifeAct::GFP is not significantly stronger than movement of MRLC::GFP of the same stage (see S4C Fig).
(TIF)

S4 Fig Preferred direction of actomyosin movement in individual follicle cells. **(A)** First row: Weighted ratios of MRLC::GFP signals moving in the Down direction (225°≤315°, retrograde) vs Up (45°≤135°, anterograde), which represent the original data shown with a *log2* scale in Fig 2C. Individual egg chambers (EC) were unified to rotate Up. The symmetry border is indicated with a red line. Box plots with medians (red) over all the analyzed follicle cells of independent egg chambers are shown. Second row: Significantly stronger MRLC::GFP retrograde movement (expressed as in A) is present during fast epithelial rotation (control stage 7) as compared to slow (stage 4) and no (*fat2* mutant of stage 1/2 and 7) epithelial rotation. *P*<0.001 (***). In contrast, no significant difference was observed when we compared MRLC::GFP retrograde movement at rotation initiation (stage 1/2) and fast epithelial rotation (control stage 7). **(B)** Weighted ratios of LifeAct::GFP signals moving in direction Down (225°≤315°, retrograde) versus Up (45°≤135°, anterograde) for follicle cells of control (stage 8) egg chambers, which were unified to rotate Up and plotted on a *log2* scale show no significant difference **(C)**, as the *P*-values indicate, to *log2* weighted ratios of MRLC::GFP movements (control stage 7 in Fig 2C and S4A Fig). **(D)** An example of a time-projected time-lapse movie that shows MRLC::GFP alignment in control (red nuclei) and *fat2* mutant (no red nuclei) follicle cells of mosaic egg chamber that contains small *fat2* mutant clones.
(TIF)

S5 Fig Manipulation of rotational speed and measurement of Myo-II pulses. **(A)** Rotational speed of analyzed egg chambers in various stages and conditions. **(B)** Rate of Myo-II intensity change (A.U.) and rate of area change (μm^2^) are shown for analyzed control (n = 28) and *fat2* mutant (n = 56) follicle cells. Individual dots represent all changes per acquired frames over time in control follicle cells (five independent egg chambers), which significantly differed from *fat2* mutant follicle cells (seven analyzed *fat2* mutant egg chambers). *P*<0.001 (***). Violin plots are shown. Black bars indicate mean. **(C)** Example comparison of representative follicle cells (control and *fat2* mutant) is shown in original units measured as MRLC::GFP intensity over time (A.U.).
(TIF)

S1 Movie Myo-II dynamics during rotation initiation in control egg chambers. Time-lapse movie of MRLC::GFP (green) signals moving at the basal surface of a young egg chamber (control stage 1/2) during rotation initiation. Membrane marker stains cell outlines (red). Frame interval = 6s. Scale bar = 5 μm. Anterior is on the left.
(AVI)

S2 Movie Myo-II dynamics during slow epithelial rotation. Time-lapse movie of MRLC::GFP (green) signals moving at the basal surface of a young slowly rotating egg chamber (control stage 4). Membrane marker stains cell outlines (red). Frame interval = 6s. Scale bar = 5 μm. Anterior is on the left.
(AVI)

S3 Movie Myo-II dynamics during fast epithelial rotation. Time-lapse movie of MRLC::GFP (green) signals moving at the basal surface of a mid-oogenesis fast rotating egg chamber (control stage 7). Membrane marker stains cell outlines (red). Note the MRLC::GFP directed movement is perpendicular to the AP axis of the egg chamber. The preferred direction against the epithelial rotation was revealed only after angular quantification (see Material and Methods and Fig 1 and Fig 2). Large MRLC::GFP dots always position towards the lagging side of follicle cells. Frame interval = 6s. Scale bar = 5 μm. Anterior is on the left.
(AVI)

S4 Movie Myo-II dynamics during rotation initiation in static egg chambers (stage 1/2). Time-lapse movie of MRLC::GFP (green) signals moving at the basal surface of a static *fat2*^*58D/103C*^ mutant egg chamber (stage 1/2) during rotation initiation. Membrane marker stains cell outlines (red). Frame interval = 6s. Scale bar = 5 μm. Anterior is on the left.
(AVI)

S5 Movie Myo-II dynamics in static egg chambers (stage 7). Time-lapse movie of MRLC::GFP (green) signals moving at the basal surface of a static *fat2*^*58D/103C*^ mutant mid-oogenesis egg chamber (stage 7). Membrane marker stains cell outlines (red). Note that MRLC::GFP is locally (within a cell) polarized (directed subcellular movement shown in Fig 2) but its planar polarity is lost (Fig 1B). Large MRLC::GFP dots were also lost. Cell outlines display deformations linked to MRLC::GFP increased intensity (Fig 4). Frame interval = 6s. Scale bar = 5 μm. Anterior is on the left.
(AVI)

S6 Movie Actin dynamics during fast epithelial rotation. Time-lapse movie of *Act5Gal4>UAS-LifeAct*::*GFP* signals at the basal surface of control stage 7–8 follicle cells. Direction of the epithelial rotation is downwards. Note that the driver used enabled patched expression of *LifeAct*::*GFP* to identify front (leading edge) actin protrusions of follicle cells. Frame interval = 6s. Scale bar = 5 μm. Anterior is on the left.
(AVI)

S7 Movie Mosaic egg chamber with small *fat2* mutant clone. Time-lapse movie showing MRLC::GFP dynamics (white) at the basal surface in *fat2*^*58D*^ mutant follicle cells (follicle cells with a lack of red nuclei) and their neighbouring controls (red nuclei) of rotating (downwards) mosaic egg chambers (stage 7/8) with small *fat2* mutant clone. Frame interval = 7s. Scale bar = 5 μm. Anterior is on the left.
(AVI)

S8 Movie Shape of follicle cells and Myo-II dynamics upon actin depletion. Time-lapse movie of MRLC::GFP (green) signals at the basal surface of a static, actin-depleted (Latrunculin A) mid-oogenesis egg chamber (control stage 7). Membrane marker stains cell outlines (red). Note that MRLC::GFP movement has ceased and MRLC::GFP large dots no longer localize to the lagging end of follicle cells. Frame interval = 6s. Scale bar = 5 μm. Anterior is on the left.
(AVI)

S9 Movie Shape of follicle cells and Myo-II dynamics upon Arp2/3 depletion. Time-lapse movie of MRLC::GFP (green) signals at the basal surface of a CK666 (Arp2/3 inhibitor) treated mid-oogenesis egg chamber of stage 7, which stalled the epithelial rotation. Membrane marker stains cell outlines (red). Note that MRLC::GFP signal movement centers as well as MRLC::GFP large dots are towards the center of follicle cells. Frame interval = 6s. Scale bar = 5 μm. Anterior is on the left.
(AVI)
