# Supplementary material for: Epithelial rotation is preceded by planar symmetry breaking of actomyosin and protects epithelial tissue from cell deformations
Source: PLoS Genet. 2017 Nov 27;13(11):e1007107. doi: 10.1371/journal.pgen.1007107 (PMC5720821; doi:10.1371/journal.pgen.1007107)
Supplement: S1 Text — (DOCX) [file pgen.1007107.s001.docx]

**Supporting Materials and Methods**

**Measurement of direction of Myo-II (small dot-like signals) movement.** To measure the direction of MRLC::GFP movement in individual follicle cells and in the plane of the analyzed epithelial tissue, we measured the angle of movement of MRLC::GFP relative to the AP axis of egg chambers (S1C Fig) with the ‘Angle’ tool in Fiji[1]. Before angle measurement, time-lapse movies were corrected for bleaching and cell membranes were registered for their movement to make them static. Angles were then measured using time (≤60s) projections of MRLC::GFP signals in time-lapse movies of 300s-600s length. No difference was observed when a 300s long time-lapse movie or a 600s long one was used for this analysis. Altogether, we measured movement of MRLC::GFP relative to the cell membrane. To unify among static egg chambers during rotation initiation (control stage 1/2), the higher value of MRLC::GFP movement identified for either Up (yellow, 45°≤135°) or Down (grey, 225°≤315°) quadrants was artificially assigned to the Down quadrant (used in Fig 1C, Fig 2C and S4A Fig). A similar approach was applied to unify among static *fat2* mutant egg chambers (stages 1/2 and 7, Fig 2B, C and S4A Fig). Ratios of retrograde to anterograde movement of MRLC::GFP and LifeAct::GFP dots (Fig 2C and S4A-C Fig) were weighted over all analyzed dots in individual cells and only cells with a minimum of 20 dots were included in this analysis.

**Measurement of Myo-II size and velocity.** The size of small and large MRLC::GFP signals was analyzed in Fiji by measuring signal diameter over 5 and 10 independent egg chambers respectively. Myo-II velocity was analyzed by measuring the displacement of Myo-II signals over 30s-60s in original time-lapse movies for 10 independent egg chambers.

**Measurement of velocity of epithelial rotation.** The velocity was defined as the average velocity over 3 independent measurements of cell membrane movement in the most central part of the confocal plane.

**Angular correction.** To define the predominant direction of the MRLC::GFP movement within follicle cells in *fat2* mutant egg chambers, we used a time-projected MRLC::GFP pattern as shown in Fig 2A. To achieve perpendicular alignment of the MRLC::GFP pattern with respect to the AP axis of an egg chamber, we then rotated the MRLC::GFP pattern of individual follicle cells through the smallest possible angle, i.e. <90°.

**Quantification of planar Myo-II and actin filament alignment in fixed tissue.** To measure the planar alignment of MRLC::GFP and actin filaments in fixed tissues, we used the Fiji software, ‘Directionality’ <http://imagej.net/Directionality>.

**Measurement of follicle cell shape and elongation direction.** Outlines of follicle cells were used to measure the roundness parameter (Shape descriptors>Roundness: 4×[*Area*]/*π*×[*Major* *axis*]^2^) in Fiji. Angles used to define the direction of elongation of follicle cells (longest axis) were measured with the ‘Angle’ tool in Fiji. The angle of Myo-II movement was measured using the time-projected MRLC::GFP pattern.

**Measurement of Myo-II intensity.** MRLC::GFP intensity was measured as the mean intensity within the defined cell outline of follicle cells. We developed the following pipeline: to measure the MRLC::GFP intensity and area of follicle cells, cell segmentation was performed using a custom macro from the IRB (Barcelona, Spain) <http://adm.irbbarcelona.org/image-j-fiji>. Afterwards, cells in consecutive time points were associated with each other if they overlapped by more than 60% according to the Jaccard index metric. Determination of mean intensity, cell area and cell roundness was done using an in-house developed plugin utilizing the Analyze Particles Plugin <http://imagej.net/Particle_Analysis> in Fiji [1]. Analyses were performed on time-lapse movies (6s frame interval), for each frame one plane in the z-axis was imaged. Bleach correction was applied in Fiji. The amplitude of the MRLC::GFP intensity change and cell area change was measured, and the mean of MRLC::GFP intensity and cell area was plotted over time (360s) for individual cells of a particular type (control vs. *fat2* mutant). Smoothed curves were then fitted, detrended, and a value (based on detrended max.-min. values) was calculated for each individual cell of a particular type and averaged over all cells of the same type. The time series were smoothened with a Gaussian filter using a window of 10 data points (i.e. 1min.) in R Studio [http://www.rstudio.com](http://www.rstudio.com/). The rate of MRLC::GFP change was calculated as the first derivative of the measured intensity. The rate of basal area reduction was calculated as the inverse value of the first derivative of the basal area.

**Cross-correlation analysis.** Cross-correlation efficiency was calculated with time shift from -6 to +6 min in R Studio. The rate of MRLC::GFP intensity change and basal area change was used to calculate the cross-correlation coefficient. The average correlation coefficient was the result of averaging the smoothed correlation data for individual cells. MRLC::GFP intensity was measured in the most central follicle cells of the focal plane of time-lapse movies.

**Measurement of planar cell chirality (PCC).** One frame of time-lapse movies was selected and the AP axis of egg chambers was turned 90° clockwise or anti-clockwise in order to unify direction of epithelial rotation to the right. In case of rotation initiation, the frame was turned 90° anti-clockwise in order to unify the anterior direction to the bottom of the frame. Oblique angle (*θ*) was measured for all cell membranes between 0°-90° (right) and -90°-0° (left) range, right and left frequencies were summarized to define to what side (left or right) cell membranes prefer to tilt as described [2].

**Supporting References**

1. Schindelin, J., et al., *Fiji: an open-source platform for biological-image analysis.* Nat Methods, 2012. **9**(7): p. 676-82.

2. Sato, K., et al., *Left-right asymmetric cell intercalation drives directional collective cell movement in epithelial morphogenesis.* Nat Commun, 2015. **6**: p. 10074.
