# Supplementary figures and images for: Epithelial rotation is preceded by planar symmetry breaking of actomyosin and protects epithelial tissue from cell deformations
Source: PLoS Genet. 2017 Nov 27;13(11):e1007107. doi: 10.1371/journal.pgen.1007107 (PMC5720821; doi:10.1371/journal.pgen.1007107)

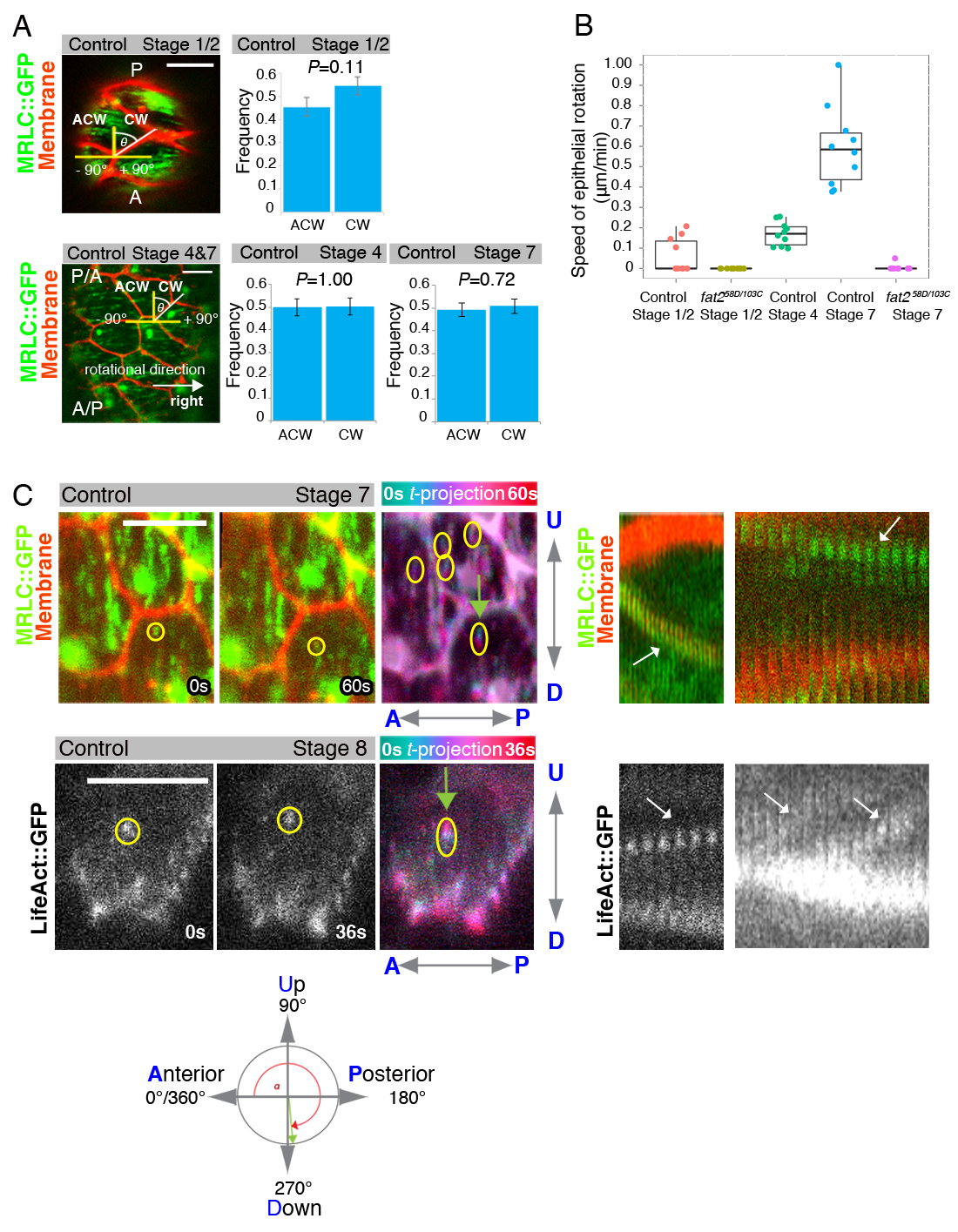

Supplement: S1 Fig — (A) Planar cell chirality (PCC) does not seem to significantly contribute to symmetry breaking during rotation initiation (stage 1/2), slow (stage 4) and fast (stage 7) rotating follicle epithelium. Number of cell membranes n = 110, n = 261 and n = 319 over 10 independent egg chambers were analyzed for rotation initiation, slow and fast epithelial rotation, respectively. S.E.M. is shown along with P values. ACW = anti-clockwise and CW = clockwise epithelial rotation, when observed from the anterior tip of an egg chamber. (B) Rotational speed (μm/min) during rotation initiation (stage 1/2), slow (stage 4), fast (stage 7) and not rotating (fat2 mutant) egg chambers (stage 1/2, stage 7) is shown. (C) Intracellular MRLC::GFP individual dot-like and LifeAct::GFP signals (example indicated with yellow circle) were analyzed at the basal surface of follicle cells. Direction of their movement (based on time-projected images, note the colour-coded t-projection, the green arrow shows the direction corresponding to almost 270° selected in yellow circle) was expressed as angle in the range of 0°-360°. Examples of stage 7/8 (fast epithelial rotation) are shown. Kymographs showing retrograde movement of MRLC::GFP and LifeAct::GFP signals (white arrows). Scale bars = 5μm. Anterior is on the left. (TIF) [file pgen.1007107.s002.tif]

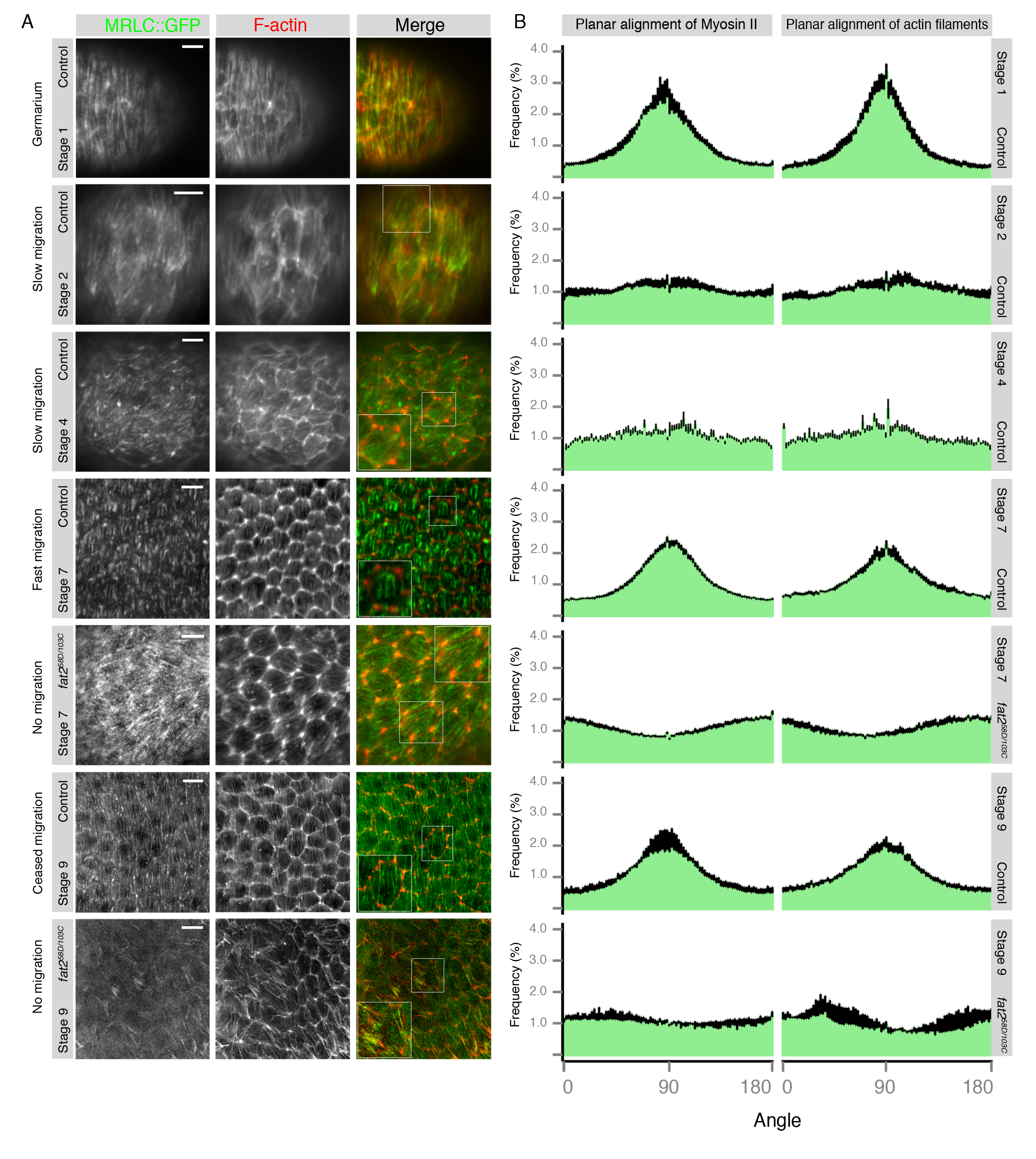

Supplement: S2 Fig — (A) Planar alignment of MRLC::GFP (green) and actin filaments (red) to the AP axis (0°-180°) at the basal side of the fixed Drosophila germarium, showing strong perpendicular alignment to the AP axis during rotation initiation, which is temporarily decreased when the egg chamber buds from the germarium (stage 2) during early oogenesis (represented by stage 4) and reaches its proper perpendicular alignment at the time of fast epithelial rotation (represented by stage 7), which is still present at stage 9 when egg chambers cease their epithelial rotation. In fat2 mutant fixed egg chambers, the MRLC::GFP planar polarized pattern was globally disturbed and reflects the direction of actin filaments at stage 7 and stage 9. White boxes show the magnification of a representative follicle cell of a particular stage, which display local MRLC::GFP signal localization. Note that MRLC::GFP displays irregular signal distribution in fat2 mutant egg chambers (stage 7 and 9) compared to corresponding controls with local MRLC::GFP asymmetric distribution during oogenesis with the epithelial rotation (stage 7). (B) Histograms represent frequency distribution of angles of MRLC::GFP movement and actin filaments (F-actin) measured between 0° and 180°. Anterior (0°) is on the left, posterior (180°) is on the right. S.E.M. is shown. Scale bars = 5μm, except of stage 9 where scale bar = 10μm. (TIF) [file pgen.1007107.s003.tif]

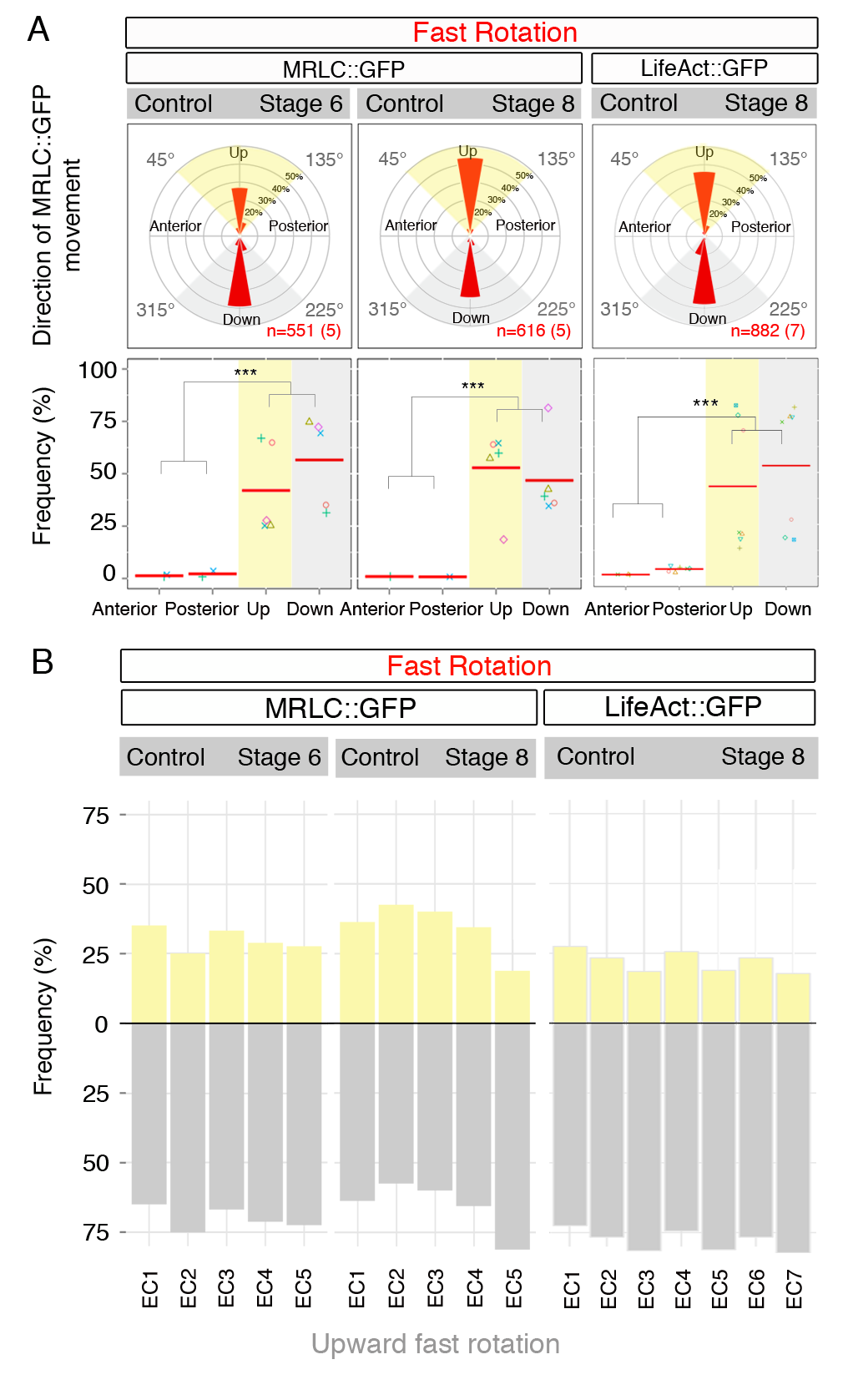

Supplement: S3 Fig — (A) First row: Angular distribution of MRLC::GFP movement expressed as frequencies plotted in 20 degree-bin rose diagrams during fast epithelial rotation (stage 6 and stage 8) is compared to LifeAct::GFP signals (stage 8). Second row: Frequencies of MRLC::GFP and LifeAct::GFP movement in four 90 degree quadrants are plotted, showing that the significant (*** = P<0.001) majority of MRLC::GFP moves within Up (yellow) and Down (grey) quadrants. The number of analyzed MRLC::GFP and LifeAct::GFP signals are indicated in red in the lower right with the number of independently analyzed egg chambers (in brackets). (B) Frequencies of MRLC::GFP movement in Up (yellow, 45°≤135°) and Down (grey, 225°≤315°) quadrants during fast (stage 6 and 8) epithelial rotation that was unified to the Up direction of epithelial rotation. Similarly, Up and Down frequencies of LifeAct::GFP movement are shown for fast epithelial rotation (stage 8) that was unified to the direction Up. Note that strong retrograde movement of LifeAct::GFP is not significantly stronger than movement of MRLC::GFP of the same stage (see S4C Fig). (TIF) [file pgen.1007107.s004.tif]

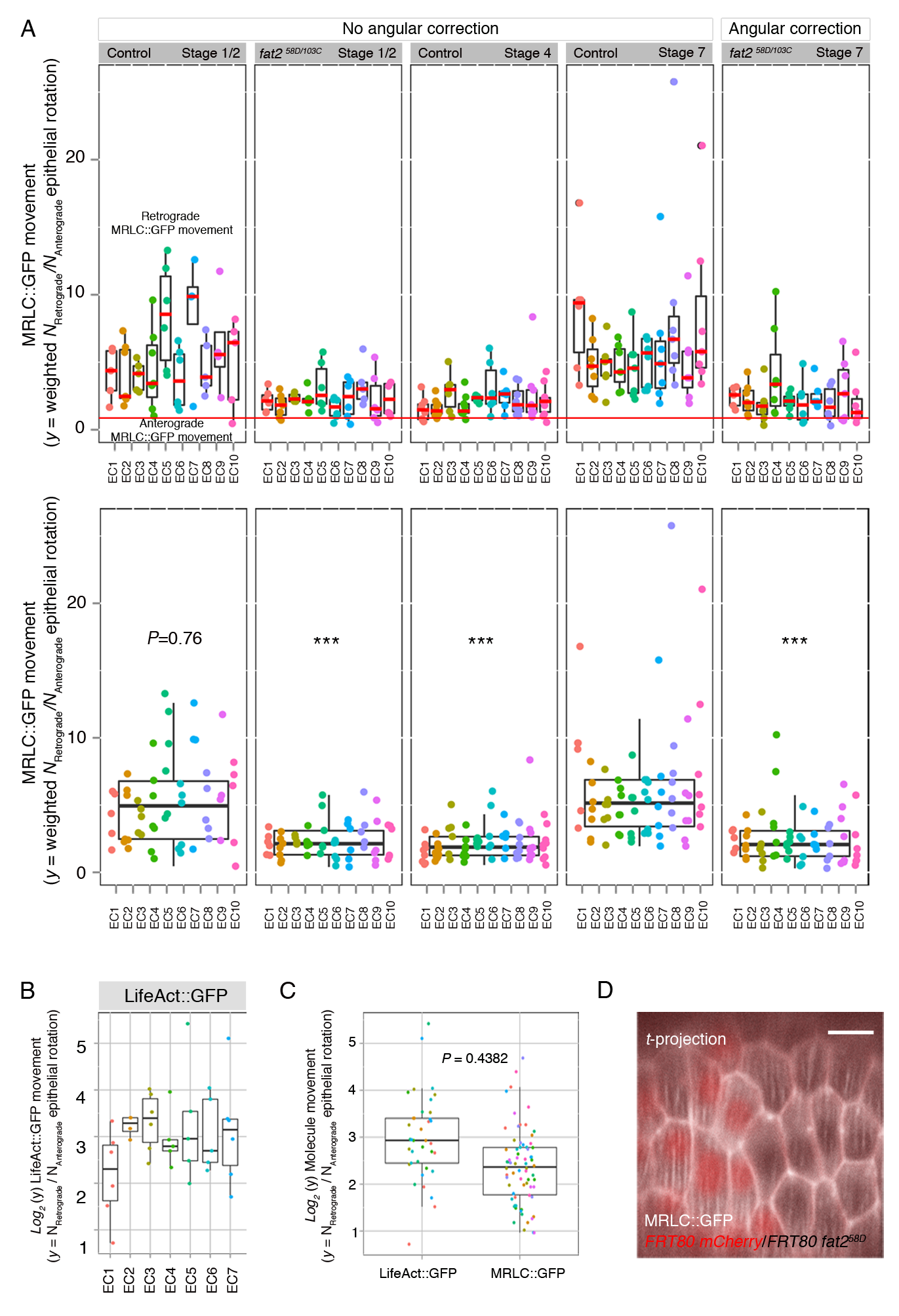

Supplement: S4 Fig — (A) First row: Weighted ratios of MRLC::GFP signals moving in the Down direction (225°≤315°, retrograde) vs Up (45°≤135°, anterograde), which represent the original data shown with a log2 scale in Fig 2C. Individual egg chambers (EC) were unified to rotate Up. The symmetry border is indicated with a red line. Box plots with medians (red) over all the analyzed follicle cells of independent egg chambers are shown. Second row: Significantly stronger MRLC::GFP retrograde movement (expressed as in A) is present during fast epithelial rotation (control stage 7) as compared to slow (stage 4) and no (fat2 mutant of stage 1/2 and 7) epithelial rotation. P<0.001 (***). In contrast, no significant difference was observed when we compared MRLC::GFP retrograde movement at rotation initiation (stage 1/2) and fast epithelial rotation (control stage 7). (B) Weighted ratios of LifeAct::GFP signals moving in direction Down (225°≤315°, retrograde) versus Up (45°≤135°, anterograde) for follicle cells of control (stage 8) egg chambers, which were unified to rotate Up and plotted on a log2 scale show no significant difference (C), as the P-values indicate, to log2 weighted ratios of MRLC::GFP movements (control stage 7 in Fig 2C and S4A Fig). (D) An example of a time-projected time-lapse movie that shows MRLC::GFP alignment in control (red nuclei) and fat2 mutant (no red nuclei) follicle cells of mosaic egg chamber that contains small fat2 mutant clones. (TIF) [file pgen.1007107.s005.tif]

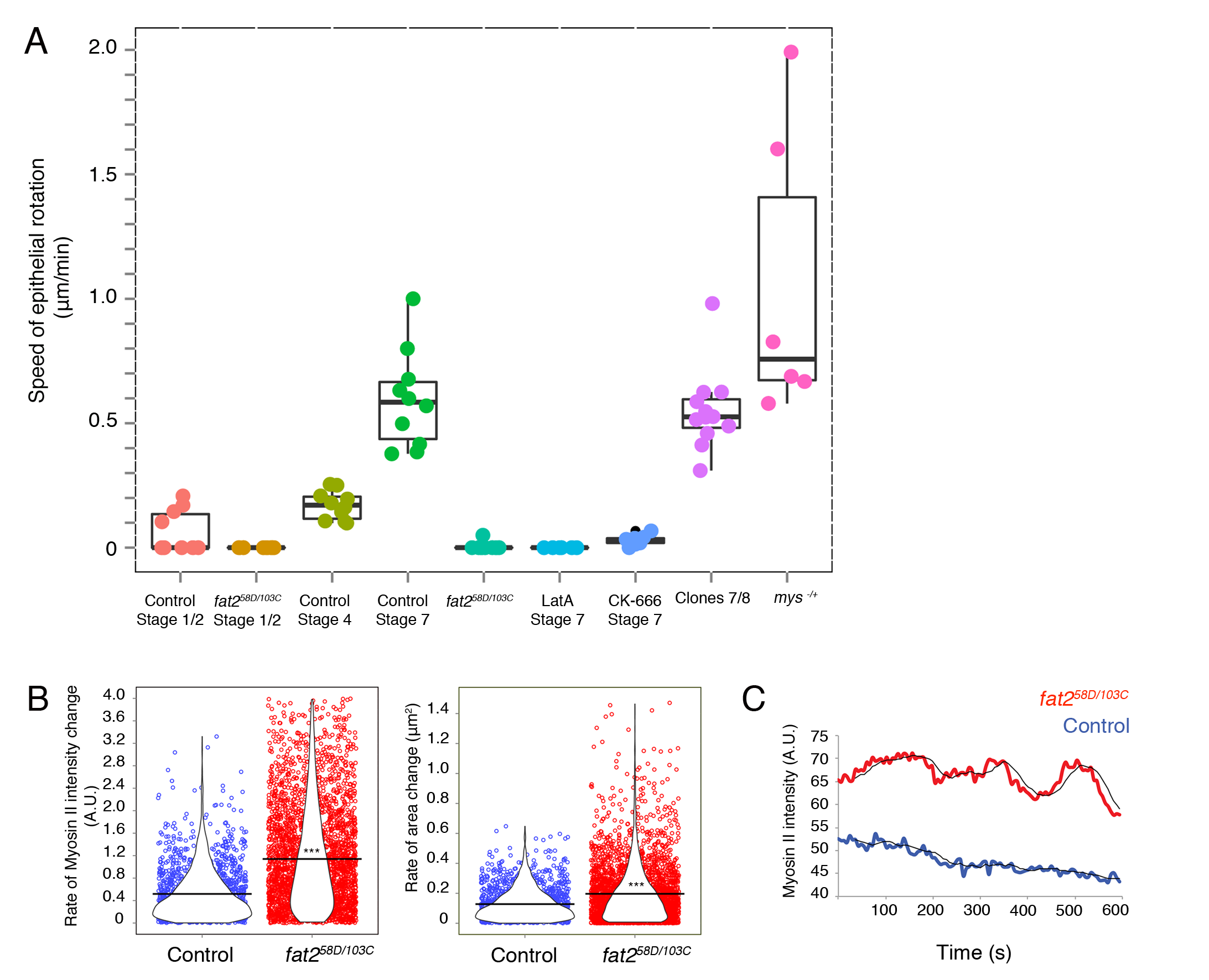

Supplement: S5 Fig — (A) Rotational speed of analyzed egg chambers in various stages and conditions. (B) Rate of Myo-II intensity change (A.U.) and rate of area change (μm2) are shown for analyzed control (n = 28) and fat2 mutant (n = 56) follicle cells. Individual dots represent all changes per acquired frames over time in control follicle cells (five independent egg chambers), which significantly differed from fat2 mutant follicle cells (seven analyzed fat2 mutant egg chambers). P<0.001 (***). Violin plots are shown. Black bars indicate mean. (C) Example comparison of representative follicle cells (control and fat2 mutant) is shown in original units measured as MRLC::GFP intensity over time (A.U.). (TIF) [file pgen.1007107.s006.tif]
